# Surgical strategies for localized colorectal amyloidosis

**DOI:** 10.1186/s40792-023-01649-0

**Published:** 2023-04-27

**Authors:** Shunya Tahara, Mohei Kohyama, Atsushi Nakamitsu, Yoichi Sugiyama, Tatsuya Tazaki, Hiroyuki Taogoshi, Toshiaki Komo, Takuro Yamaguchi, Mitsuharu Ueda, Akira Ishikawa, Shinya Takahashi, Masaru Sasaki

**Affiliations:** 1 Department of Surgery, JA Hiroshima General Hospital, 1-3-3 Jigozen, Hatsukaichi, Hiroshima 738-8503 Japan; 2 Department of Neurology, Graduate School of Medical Sciences, Kumamoto University, 1-1-1 Honjou, Chuo Ward, Kumamoto, Kumamoto 860-8556 Japan; 3 Department of Molecular Pathology, Graduate School of Biomedical and Health Sciences, Hiroshima University, 1-2-3 Kasumi, Minami Ward, Hiroshima, Hiroshima 734-0037 Japan; 4 Department of Surgery, Graduate School of Biomedical and Health Sciences, Hiroshima University, 1-2-3 Kasumi, Minami Ward, Hiroshima, Hiroshima 734-0037 Japan

**Keywords:** Localized colorectal amyloidosis, Segmental type, Pan-colon type

## Abstract

**Background:**

Localized colorectal amyloidosis has a good prognosis, but cases involving bleeding or perforation may require surgery. However, there are few case reports discussing the differences in the surgical strategy between the segmental and pan-colon types.

**Case presentation:**

A 69-year-old woman with a history of abdominal pain and melena was diagnosed with amyloidosis localized in the sigmoid colon by colonoscopy. Since preoperative imaging and intraoperative findings could not rule out malignancy, we performed laparoscopic sigmoid colectomy with lymph-node dissection. Histopathological examination and immunohistochemical staining revealed a diagnosis of AL amyloidosis (*λ* type). We diagnosed localized segmental gastrointestinal amyloidosis, because there was no amyloid protein in the margins, and the tumor was localized. There were no malignant findings.

**Conclusions:**

Unlike systemic amyloidosis, localized amyloidosis has a favorable prognosis. Localized colorectal amyloidosis can be classified into the segmental type, in which amyloid protein is deposited locally, and the pan-colon type, in which amyloid protein is deposited extensively in the colon. Amyloid protein causes ischemia due to vascular deposition, weakening of the intestinal wall due to muscle layer deposition, and decreased peristalsis due to nerve plexus deposition. No amyloid protein should remain outside the resection area. The pan-colon type is often reported to cause complications such as anastomotic leakage, and primary anastomosis should be avoided. On the other hand, if there is no contamination or tumor remnants in the margin, the segmental type may be considered for primary anastomosis.

## Background

Amyloidosis is characterized by extracellular deposition of amyloid protein in various organs [1]. Amyloid proteins consist mainly of insoluble fibrous proteins with a beta-sheet structure. To date, 36 amyloid proteins have been identified in humans. Immunoglobulin amyloid light-chain (AL) amyloid is derived from monoclonal immunoglobulin light chains produced by abnormal plasma cells [2]. AL protein is often deposited in the heart, kidneys, soft tissues, liver, nerves, and gastrointestinal tract, causing dysfunction in the deposited organs [3]. Systemic amyloidosis with amyloid deposits in multiple organs has a poor prognosis, but localized amyloidosis involving only one organ has a good prognosis [4]. Localized gastrointestinal amyloidosis can be classified into the pan-colon type, in which amyloid protein is distributed relatively widely throughout the gastrointestinal tract, and the segmental type, in which amyloid protein is deposited locally [5]. We experienced a surgical case of localized segmental amyloidosis of the sigmoid colon, but there is virtually no literature on the surgical strategies for such a case. In this case report, we discuss the surgical strategies for amyloidosis, with a review of other reported cases.

## Case presentation

**Fig. 1:**
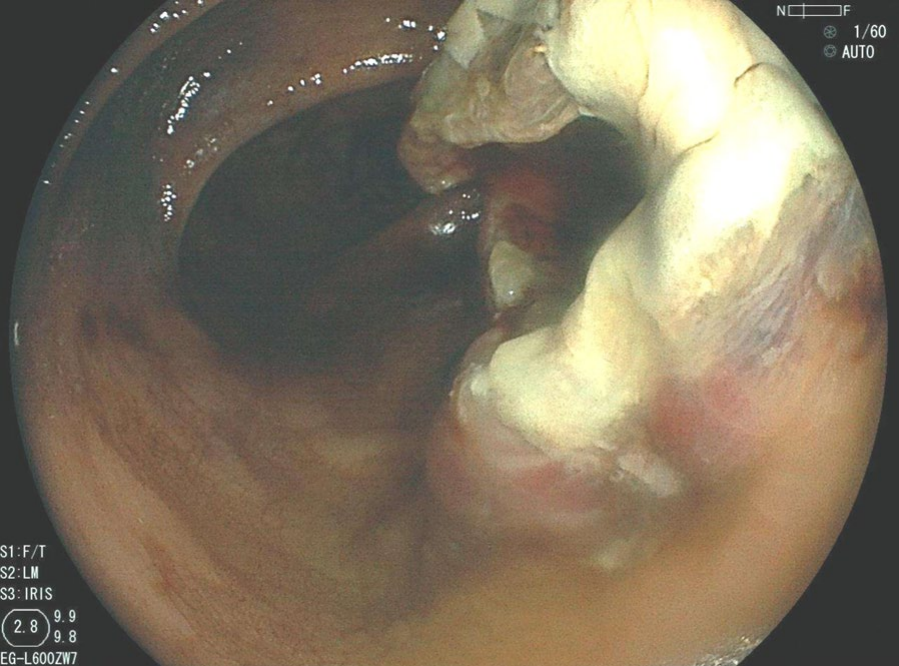
Lower gastrointestinal endoscopy revealed a localized elevated submucosal tumor-like lesion with a deep ulcer in the center of the sigmoid colon 20 cm from the anal verge

**Fig. 2:**
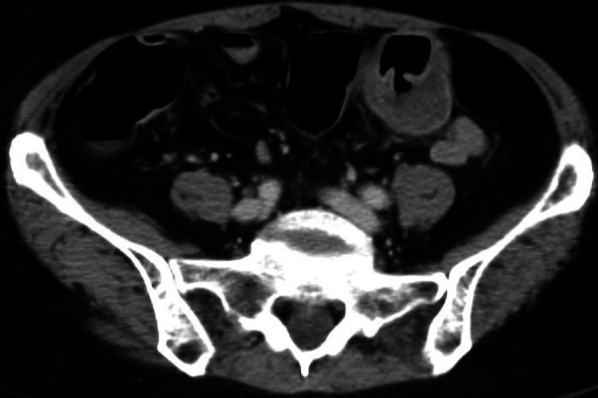
Contrast-enhanced computed tomography revealed a soft tissue with a poor central contrast effect localized to the sigmoid colon. The tumor had a depression suspected to be an ulcer. There was no evidence of surrounding tissue infiltration, free air, or ascites

A 69-year-old Japanese woman with a history of hypertension was referred to the hospital for abdominal pain and melena. No shock was evident, and lower gastrointestinal endoscopy revealed a well-defined submucosal tumor-like protruding lesion with a central ulcer in the sigmoid colon (Fig. 1). The lesion was localized to the sigmoid colon. A localized submucosal tumor-like elevated lesion, redness, submucosal hemorrhage, and ulcer formation, which are typical findings of AL amyloid protein deposition, were not observed in other parts of the colon, so the lesion was not diagnosed as the pan-colon type. Biopsy results revealed a non-epithelial tumor with positive Dylon and Congo-red staining, leading to the diagnosis of amyloid tumor. Contrast-enhanced computed tomography showed a localized soft-tissue mass with a poor internal contrast effect in the sigmoid colon (Fig. 2). There was no organ invasion or perforation, no swollen lymph nodes, and no distant metastasis. Serum levels of the tumor markers carcinoembryonic antigen (1.7 U/ml, normal range < 5.0 U/ml) and carbohydrate antigen 19–9 (6.1 U/ml, normal range < 37.0 U/ml) were within the normal ranges. Proteinuria and an increased serum creatinine level were not observed before surgery. No signs suggestive of heart failure were observed based on the clinical, ECG, and chest X-ray findings before surgery. In addition, there were no signs of amyloid protein deposits in other organs, and the diagnosis was localized colorectal amyloidosis.

**Fig. 3:**
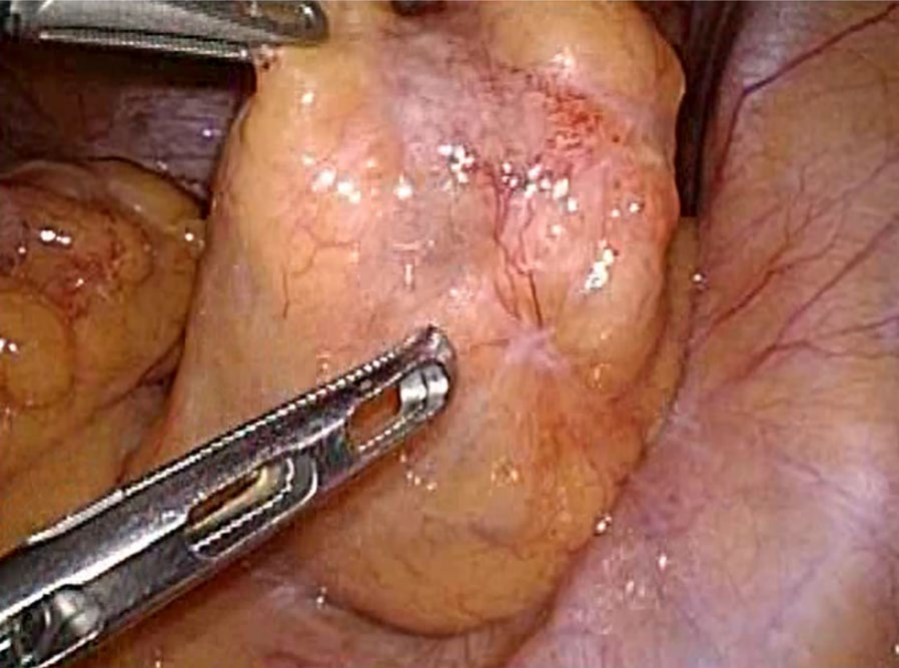
Intraoperative findings revealed a soft tissue in the sigmoid colon with an uneven serosal surface

At the time of surgical referral, the abdominal pain had improved, and tenderness and peritoneal irritation signs were absent. The gastrointestinal amyloidosis was localized but symptomatic, so the patient underwent elective laparoscopic sigmoid colectomy as well as a total biopsy. There was a soft mass in the sigmoid colon, and the serosal surface was slightly uneven (Fig. 3). There were no other tumors. Preoperative imaging and intraoperative findings did not exclude the possibility of malignancy, and D3 dissection was performed. The colon was anastomosed using a double staple technique. There were no intraoperative complications.

**Fig. 4:**
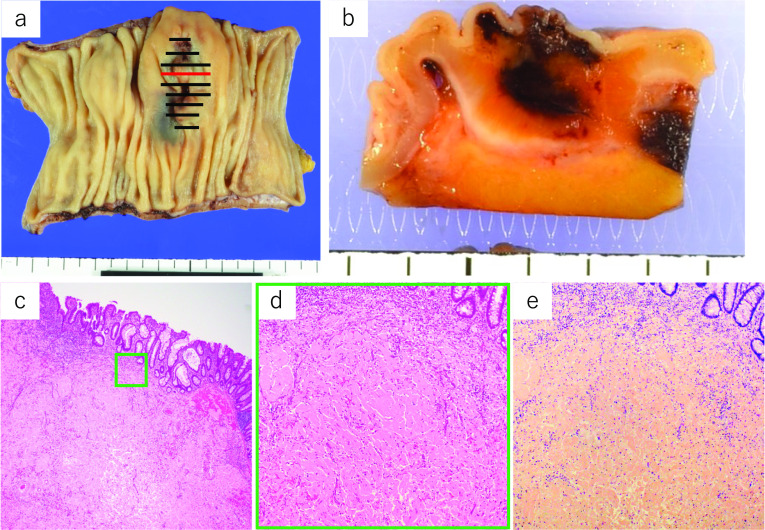
Macroscopic findings indicated a 35 × 30 mm submucosal tumor-like lesion in the sigmoid colon. The black and red solid lines indicate the deposition of amyloid protein (**a**). There was no amyloid protein in the margins. A sliced section showed a poorly circumscribed, yellowish lesion with hemorrhage within the colon wall (**b**). Histopathological findings indicated that the epithelial integrity of the mucosa was preserved, and eosinophilic material was present in the submucosal and deeper layers (hematoxylin and eosin staining × 40) (**c**). Eosinophilic material was structureless (H&E × 100) (**d**). This tumor was positive for Congo-red staining (× 100) (**e**)

**Fig. 5:**
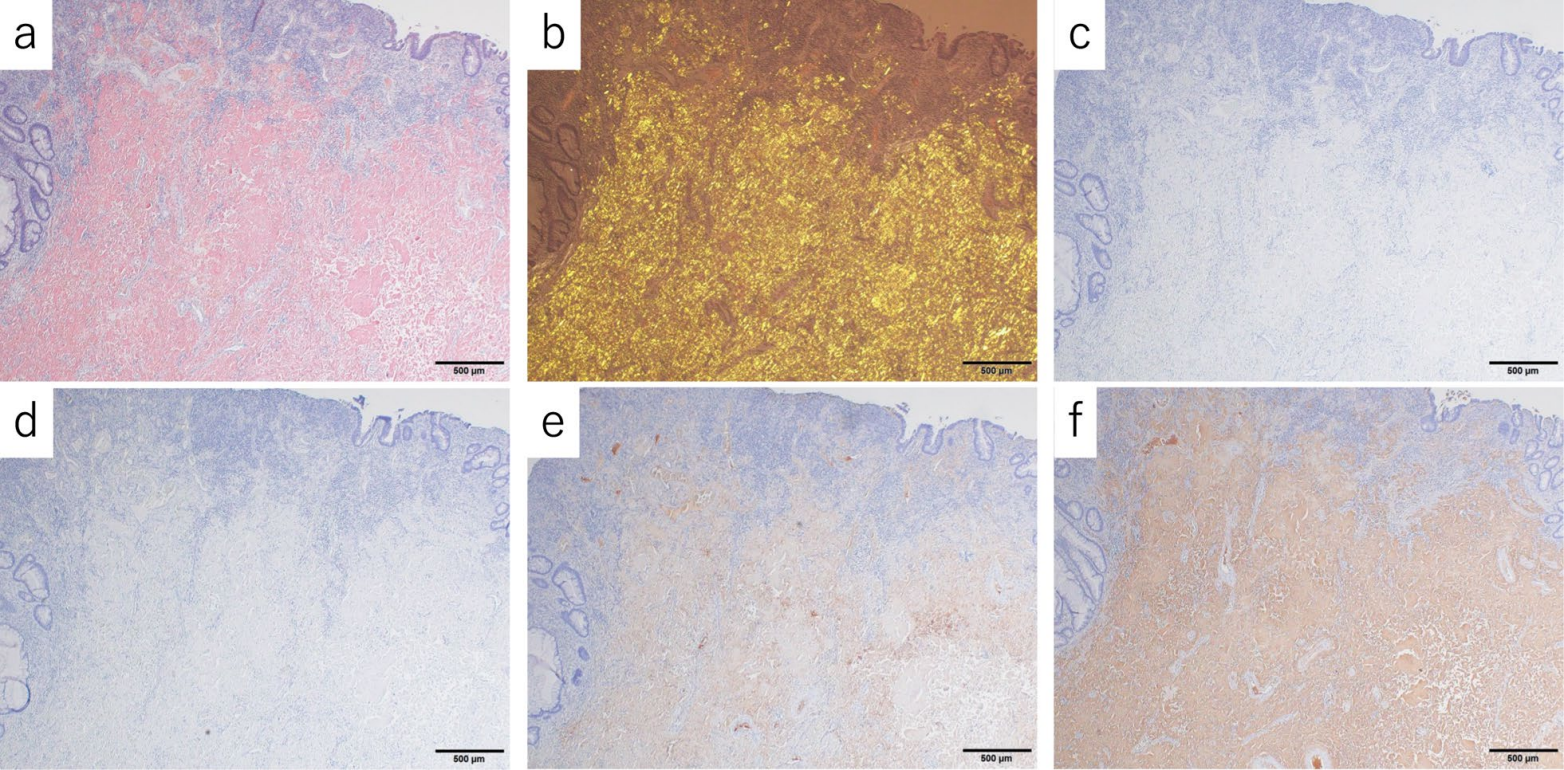
The tumor was positive for Congo-red staining (**a**) and green polarized light on polarized light microscopy (**b**). Immunohistochemically, the amyloid protein was negative for amyloid A (**c**) and transthyretin (**d**), but positive for amyloid light-chain protein. In particular, the sites of amyloid deposition were not so much consistent with the *κ* type (**e**) as with the *λ* type (**f**)

Postoperatively, the patient had chylous ascites, which resolved with conservative treatment consisting of only a fat-restricted diet. The patient was discharged from the hospital on postoperative day 10. Histopathologic examination revealed positive Dylon and Congo-red staining, mainly in the submucosa to subserosa, consistent with a tumor, as well as deposition of acidophilic inorganic material with green polarized light on polarized light microscopy (Fig. 4). Immunohistochemical staining revealed AL amyloidosis (*λ* type) (Fig. 5). As there was no amyloid protein in the margins, we diagnosed a localized gastrointestinal amyloid tumor. The submucosal tumor-like protruding lesion was caused by massive deposition of amyloid protein in each layer, and the ulceration was probably caused by ischemic changes due to occlusion of the blood vessel lumen. Reactive atypia of the mucosa due to ulceration and erosion was observed, but there was no evidence of malignancy or significant lymph-node involvement. She was alive without recurrence at 9 months after the operation; lower gastrointestinal endoscopy showed no anastomotic abnormalities, and there were no findings in other mucosae suspected to have amyloid deposition.

## Discussion

AL amyloidosis is the most frequent type of amyloidosis compared with other types, and the annual incidence of new cases of amyloidosis is high (78%) [3]. The unadjusted prevalence of AL amyloidosis was 40.5 cases per million person-years in the United States in 2015 [6]. Different amyloid proteins tend to be deposited at different sites, with AL amyloidosis being particularly prone to deposition in the muscularis mucosa, submucosa, and muscularis propria [7, 8]. If amyloid protein is present in the mucosa, submucosa, or intrinsic muscle layer, localized raised or tumor-like lesions may be seen grossly and may present symptoms of intestinal obstruction [9]. High levels of amyloid protein can also be deposited in the vascular wall and can impair blood flow, inducing erythema, submucosal hematoma, ulceration, and perforation [10]. The prognosis of systemic AL amyloidosis is poor [3], but there are case reports of localized amyloidosis that did not progress to systemic amyloidosis and responded well to treatment [11–13]. However, amyloidosis with perforation and hemorrhage can have a fatal outcome. Surgery may be a treatment option if bleeding, perforation, or complications of malignancy are suspected.

In the literature published to date, 14 cases of surgery for colorectal amyloidosis have been reported [14–27]. Excluding one case in which the extent of amyloid deposition was unknown [24], these cases comprised four systemic amyloidosis [14, 18, 21, 22] and nine localized amyloidosis cases [15–17, 19, 20, 23, 25–27]. Among the localized amyloidosis cases, four were the pan-colon type [15, 17, 25, 26] and five were the segmental type [16, 19, 20, 23, 27]. Among the pan-colon type cases, three received primary anastomosis [15, 17, 26], while two had recurrent gastrointestinal symptoms and underwent additional treatment [17, 26]. The remaining case in which anastomosis was not performed also had to be reconstructed due to repeated stenosis caused by amyloid deposition in the colostomy [25]. Therefore, three of the four pan-colon type cases had postoperative complications due to amyloid deposition. Of the segmental type cases, excluding two in which Hartmann's operation was performed due to perforation or obstructive enterocolitis [20, 23], primary anastomosis was performed in three cases [16, 19, 27]. One patient experienced postoperative suture failure [19], but the other four had no specific postoperative complications.

Three changes result from amyloid protein deposition in the intestinal wall. The first is the deposition of amyloid protein in the vessel wall, which causes vessel wall thickening and weakening. These changes result in narrowing of the vessel lumen and infarction, leading to ischemia of the intestinal wall. Second, amyloid protein deposited within the intestinal wall destroys the muscle layer, resulting in weakening of the intestinal wall. Third, deposition of amyloid protein in the Auerbach's plexus leads to decreased peristalsis. Decreased peristalsis causes intestinal contents to become stagnant, resulting in increased intestinal pressure [28]. This change can lead to not only gastrointestinal perforation but also postoperative suture failure; thus, the decision to anastomose the intestinal tract during surgery for gastrointestinal amyloidosis is controversial.

In the Japanese literature, there was a case of three times of colonic perforations that eventually revealed amyloid protein deposition in the entire colon, and the diagnosis was the pan-colon type of localized amyloidosis [29]. Ussia et al. also reported a case of stoma reconstruction for repeated stoma stenosis after Hartmann surgery for colonic perforation and found amyloid deposits in the additionally resected intestinal tract [25]. Because the pan-colon type has many postoperative complications, easy primary anastomosis should be avoided, and sub-total colectomy should be considered. On the other hand, the segmental type may be considered for primary anastomosis in the absence of contamination or an abnormal intestinal stump.

In this case, we determined from the preoperative pathology and intraoperative findings that the patient had localized segmental amyloidosis, and primary anastomosis was performed. The postoperative pathology also supported the results. As far as we could find, there are no studies mentioning the safety of anastomosis or resection margins in the segmental type of localized amyloidosis. Therefore, it is important to consider the segmental type or pan-colon type in planning treatment strategies preoperatively. However, unlike cancers arising from the mucosa, in gastrointestinal amyloidosis, it is difficult to determine the exact extent of amyloid deposition preoperatively based on endoscopic findings and tissue biopsy alone. Because of the autofluorescence property of the AL amyloid protein, Kuroha et al. mentioned the possibility that areas of amyloidosis will appear bright green on autofluorescence endoscopy [30]. This method may be more useful for confirming the extent of amyloid deposition.

## Conclusions

Localized colorectal amyloidosis has a good prognosis but may require surgery due to perforation or bleeding. The segmental type may be considered for primary anastomosis. On the other hand, sub-total colectomy should be considered as an option, and primary anastomosis should not be performed in the pan-colon type.

## Data Availability

All the data and materials used in this study were obtained from publicly available sources or databases, and all cited literature is accessible through PubMed.

## Abbreviation

AL: Amyloid light-chain
